# Removing Disperse red 60 and Reactive blue 19 dyes removal by using *Alcea rosea* root mucilage as a natural coagulant

**DOI:** 10.1186/s13568-019-0839-9

**Published:** 2019-07-22

**Authors:** Tahereh Zarei Mahmoudabadi, Parvaneh Talebi, Mahrokh Jalili

**Affiliations:** 1 Department of Environmental Health Engineering, Shahid Sadoughi University of Medical Science, Yazd, Iran; 2 Department of Environmental Health Engineering, Shahid Sadoughi University of Medical Science, Abarkouh Paramedical School, Yazd, Iran

**Keywords:** Dye removal, Sewage treatment, Natural coagulant, *Alcea rosea*

## Abstract

In terms of health, dyes have carcinogenic, mutagenic and toxic properties and can have adverse effects on health and the environment. Therefore, sewage containing to dyes must be purified before being discharged into the environment. The current study aimed to investigate the effectiveness of *Alcea rosea* root extract in Disperse red 60 and Reactive blue 19 dyes removal from synthetic sewage. In this study, the effect of different indices including pH (5–11), *Alcea rosea* concentration (50–300 mg/L) and initial dye concentration (10–80 mg/L) was investigated. During the tests, the coagulant was stirred with rapid mixing at a speed of 250 rpm for 2 min. In the following, the speed (30–60 rpm) and the time (10–25 min) were used for slow mixing and after mixing the effect of settling time (10–60 min) and temperature (20–70) on removal efficiency of Disperse and Reactive dyes was investigated. The results showed that the maximum of removal efficiency of Disperse and Reactive dyes in optimum conditions including (pH = 11, coagulant concentration = 200 and 250 mg/L, dye concentration 40 and 20 mg/L, speed 60 rpm, during 15 min with settling time 60 min and temperature 60 °C obtained 86% and 68%, respectively. According to the result, the *Alcea rosea* coagulant has the best ability in removing dyes from aqueous solutions and sewage, especially Disperse dyes. Disperse dye is much eliminated in the coagulation process due to its lower solubility, higher suspending materials and less required solved chemical oxygen demand to the total chemical oxygen demand (SCOD/TCOD).

## Introduction

In recent years, development of industries has been caused environmental pollution because there are organic and inorganic pollutants, heavy metals, dyes, etc.in their sewage from production process (Santhi et al. 2016). Due to the complicated and non-biodegradable molecular structure, dye is the most important pollutants in the industries, that can’t be removed by biological methods (Cengiz et al. 2012). But the chemical purification procedure can be useful in the removal of this contaminant (Chen et al. 2018). Annual production of dyes is estimated at more than 700,000 tones (Moussavi and Mahmoudi 2009). The presence of organic dyestuffs in the wastewater prevents the penetration of light into the receiving waters, disrupting in the photosynthesis of living aquatic organisms in these resources, and reducing the transfer of oxygen into the water. Also increasing the toxic effects of the accumulation of pollutants causes irreparable damage to the environment (Haque et al. 2015; Yu et al. 2009) and dangerous to public health due to adverse health and environmental impacts (Tie et al. 2015). Discharging these wastewaters into rivers and lakes without any treatment makes a reduction in the quality of surface and underground water resources (Raman and Kanmani 2016).

Three dyes that commonly used in industries, and based on their chemical nature are classified as follows: acidic, Reactive and Disperse Dyes. Acidic dyes are water-soluble and Anionic compounds that used for nylon, wool, and silk fibers (Phalakornkule et al. 2010). Disperse dyes are part of the derivatives of nitro-phenyl amine and azoanthraquinone dyes and don’t contain sulfated groups. Disperse dyes are soluble in yarn but insoluble in water (El-sayed et al. 2012). Reactive dyes are anionic, soluble in water and very stable. Due to circular aromatic structure, have carcinogenic and mutagenic in term of health and can make allergies and skin problems (Al-Momani et al. 2002). In recent years, the idea of economic savings has become a necessity, so designing some methods for removing contaminants should be based on using materials that can remove these pollutants at the lowest cost. Recently, the methods of using natural materials like plant extracts as coagulants that are biodegradable and low cost have provided favorable result in the direction of wastewater treatment in combination with other materials (Kasprzyk and Gajewska 2018). One of these methods for wastewater treatment is coagulation and flocculation, which is widely used in the initial treatment of sewage (Zarei Mahmudabadi et al. 2018). There are various processes for removing of dyes from industrial wastewater that can point to processes such as biological process, chemical Oxidation, Adsorption, Photo catalyst, Electrocoagulation, Coagulation, and Flocculation (Yagub et al. 2014; Zodi et al. 2013; Guimarães et al. 2012; Gupta et al. 2012; Verma and Dash 2012; Wang et al. 2009). In the meantime, using natural coagulants is more widely accepted because of its biodegradability, low cost, and lack of residual material in the wastewater (Maurya and Daverey 2018).

Natural coagulants that is used in other studies include the chitosan, moringa seeds, banana skin powder, scrotum powder, banana stalk hair, papaya seed powder, etc. (Vilaseca et al. 2014; Veeramalini et al. 2012). One of these natural coagulants is *Alcea rosea* root extract that their effectiveness on Disperse and Reactive dyes removal hasn’t been investigated, yet.

*Alcea rosea*, the common hollyhock, is an ornamental plant in the *Malvaceae*. In herbal medicine, it is believed the Hollyhock is an emollient and laxative. It is used to control inflammation, to stop bedwetting and as a mouthwash in cases of bleeding gums. The flowers are a range of dyes from white to dark red, including pink, yellow and orange. Different dyes prefer different soils. It was imported into Europe from southwest of China during, or possibly before, 15th century. The flowers and leaves have about 6 to 9 percent of mucilage that the highest percentage of which is slightly before flowering. Also, flavonoids and a very small amount of essential oils are from other flowers and leaves. The most important *Alcea* root composition is its mucilage, which varies in different seasons and reaches the maximum in winter. Different sources mention the percentage of root Mucilage between 10 and 35%. Mucilage induces rhamnose, galactose, and galacturonic acid through hydrolysis (Ahmadi et al. 2014; Shah et al. 2011).

So far, multiple studies have been done on removing Disperse and Reactive dyes using natural coagulants, which are coated by *Ocimum basilicum* for textile wastewater treatment (Shamsnejati et al. 2015), natural zeolite modified with phosphoric acid and sulfuric acid (Maleki et al. 2013), *Plantago major* extract as a natural coagulant in removal of Reactive blue 19 dye (Zarei Mahmoudabadi et al. 2019) and natural coagulant in removing other dyes including reactive yellow 2 dye (Veeramalini et al. 2012), effective performance of chitosan, Surjana seed powder and corn seed powder as a natural coagulant in removing Congo red dye from aqueous solutions (Patel and Vashi 2012) and they have had a good performance.

However, due to advantages such as ease of use, naturalness, availability, and possibility of producing secondary compounds less than chemical methods, using Alcea root mucilage can be used as a coagulant in the removal of sewage containing dyes. This study aimed to investigate the removal of Disperse red 60 and Reactive blue 19 dyes from synthetic sewage by coagulation process and by using *Alcea rosea* root mucilage as a natural coagulant.

## Materials and methods

### Preparation of *Alcea rosea* mucilage extract

**Fig. 1:**
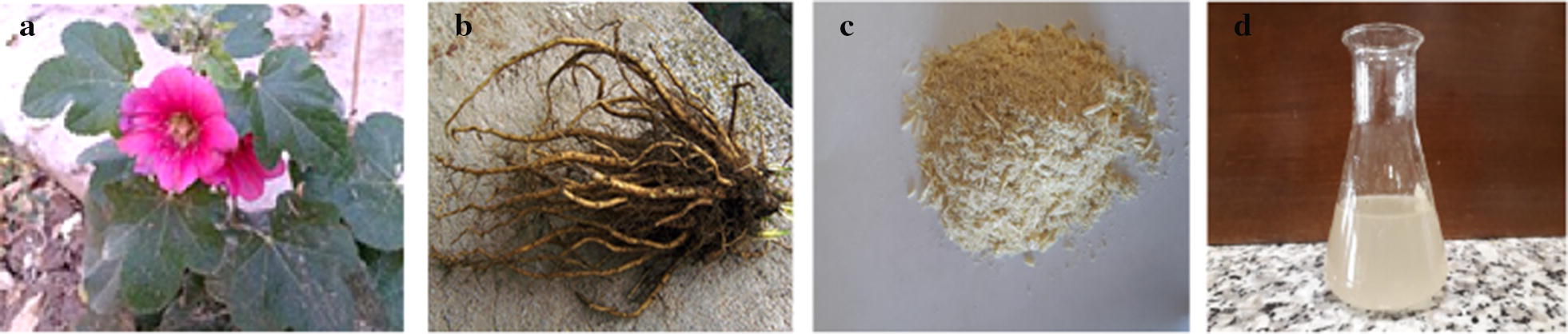
Used species (**a**), root (**b**), powder (**c**), mucilage (**d**) of *Alcea rosea* in this study

Alceahas 60 species, which *Alcea rosea* was used in the current study (With pink flowers) (see Fig. 1). To prepare, the root were naturally dried and then milled. Then, 2.5 g of dried root powder was added to 100 mL of 0.5 M sodium chloride which was prepared with distilled water, and the resulting solution was shaken for 1 h by using a magnetic magnet (Subramonian et al. 2014). In this study, a specific mesh for coagulant powder was not used. 1 g of coagulant root after milling was used directly for mucilage extraction.

The resulting milky solution mucilage was passed through a fabric filter and used. This action was carried out to extract and activate the coagulation components. 25 g/L of storage milky solution of *Alcea rosea* root mucilage is prepared on a daily basis. This solution was investigated as a coagulant in this study.

### Measurement of dye removal

In this study the physical and chemical properties of the used dyes are shown in Table 1.

**Table 1:** Basic properties of the investigated dyes (Siddique et al. 2011; El-sayed et al. 2012)

Characteristic	Dispersion red 60	Reactive blue 19 dyes
Chemical formula	C_20_H_13_NO_4_	C_22_H_16_N_2_Na_2_O_11_S_3_
Molecular weight	331.32 g/mol	626.55 g/mol
CAS	17418-85-5	2580-78-1
C.I	60,756	61,200
Molecular structure		

Measurement of dyes removal was performed by using absorption measurement. In this way at first, a solution of dye at a concentration of 1000 mg/L was prepared. Standard solutions were prepared at concentrations of 5, 10, 20, 40, 60, 80 and 90 mg/L. With the DR6000 spectrophotometer, (HACH), the adsorption rate for Disperse red 60 and Reactive blue 19 at wavelength of 586 nm and 592 nm obtained, respectively. Calibration curve was drawn. The initial dye concentration was determined by using a calibration curve. The percentage of dye removal was calculated by using the below equation:

$${\text{Dye removal }}\left( {\text{\%}} \right) = \frac{{\left( {{\text{Abs}}_{0} - {\text{Abs}}} \right)}}{{{\text{Abs}}_{0} }} \times 100$$

${\text{Abs}}_{0}$ is the dye absorption mean before treatment and Abs is the dye concentration after the treatment process.

This empirical study was conducted on a laboratory scale in the water and wastewater chemistry laboratory of the Faculty of Health of Yazd University of Medical Sciences.

After coagulant preparation, 100 mg/L was added to 1000 mL of dyeing solution at a concentration of 40 mg/L with different pH (5–10). PH adjustment was performed by using sodium hydroxide and one chloride acid (manufactured by Merck GmbH) and a pH meter (HAC multiparameter manufactured by the HQ40 model). After mixing with Jarrett (manufactured by HACH USA, model 402-7790), the settling time about 60 min were given to the solution, and then samples were collected from 3 cm below the sewage surface using pipettes. By measuring the removal efficiency of the sample, the optimum pH for coagulant was determined. Subsequently, considering the optimal pH and constant dye concentration, different doses of *Alcea rosea* (50, 100, 150, 200, 250 and 300 mg/L) were added to the sample. By measuring the removal efficiency of the paint, the optimal dose of *Alcea rosea* was determined for both disperse and reactive dyes. In the next step, with the optimal conditions obtained from the previous steps, the effects of different concentrations of dye (10, 20, 40, 60 and 80 mg) were investigated. During the tests, the coagulant was stirred at a speed of 250 rpm for 2 min. These values were kept constant for all experiments. Then, various speeds (30, 40 and 50 rpm) were investigated for 10, 15, 20 and 25 min to slow mixing. Finally, having optimum conditions that obtained from the previous steps, the effect of different sedimentation times (10, 20, 30, 40, 50 and 60 min) were investigated after finishing the mixing process.

It should be noted that at all steps of the experiments were carried out at 40 °C. Finally, the effects of different temperatures (20, 30, 40, 50, 60 and 70 °C) were investigated for optimal temperature determination.

## Results

### PH effects

**Fig. 2:**
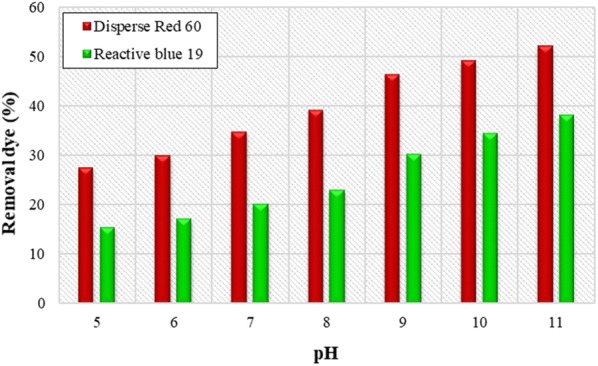
The effect of initial pH on the efficiency of removal of Disperse red 60 and Reactive blue 19 dyes. (*Alcea rosea* = 100 mg/L; initial dye concentration = 40 mg/L)

PH of the sample plays an important role in the process of coagulation and flocculation, so that each coagulant in a particular pH can have a significant effect. Figure 2 shows the effect of *Alcea rosea* coagulant in dye removal in different pH. The results indicate that pH = 11 is the most appropriate pH. So that removal efficiency for *Alcea rosea* coagulant at pH = 11 was 55.3% and 43.4% respectively for Reactive and Dispersive dyes. Then all experiments were performed at optimal pH.

### The effect of coagulant dose

**Fig. 3:**
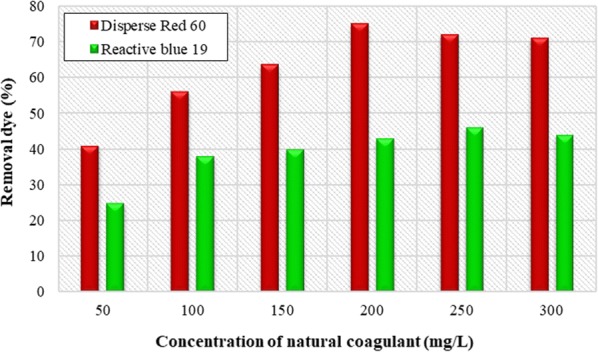
Effect of *Alcea rosea* concentration on removal efficiency of Disperse red 60 and Reactive blue 19 dyes. (Initial pH = 11 and initial dye concentration = 40 mg/L)

The optimal concentration of *Alcea rosea* coagulant, as a natural coagulant, was determined for Disperse and Reactive dyes removal at optimal pH for each sample (Fig. 3). As it can be seen, with increasing in the amount of natural coagulant, the amount of dye removal increased. The maximum removal of Disperse and Reactive dyes were 75% at 200 mg/L and 46% at 250 mg/L of *Alcea rosea* root mucilage, respectively.

### The effect of initial dye concentration

**Fig. 4:**
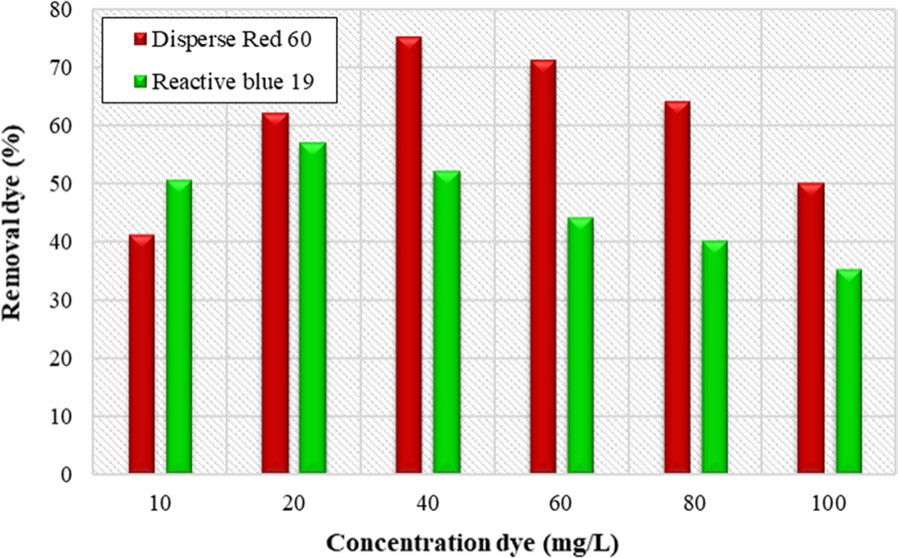
Effect of initial dye concentrations on removal efficiency of Disperse red 60 and Reactive blue 19 dyes. (Initial pH = 11 and *Alcea rosea* concentration = 200 mg/L for Disperse experiments; initial pH = 11 and concentration = 250 mg/L for Reactive experiments)

Figure 4 shows removal efficiency of dye in different concentrations of the dye under obtained optimal conditions from the previous steps (pH and coagulant concentration). The results showed that the highest removal of Disperse dye with 75% efficiency was obtained at 40 mg/L of dye concentration. The maximum Reactive dye removal efficiency was also achieved with 56.8% efficiency at a concentration of 20 mg/L. In the following experiments, concentration of 40 mg/L and 20 mg/L of Dispersion and Reactive dyes were used, respectively.

### The effect of slow mixing speed

**Fig. 5:**
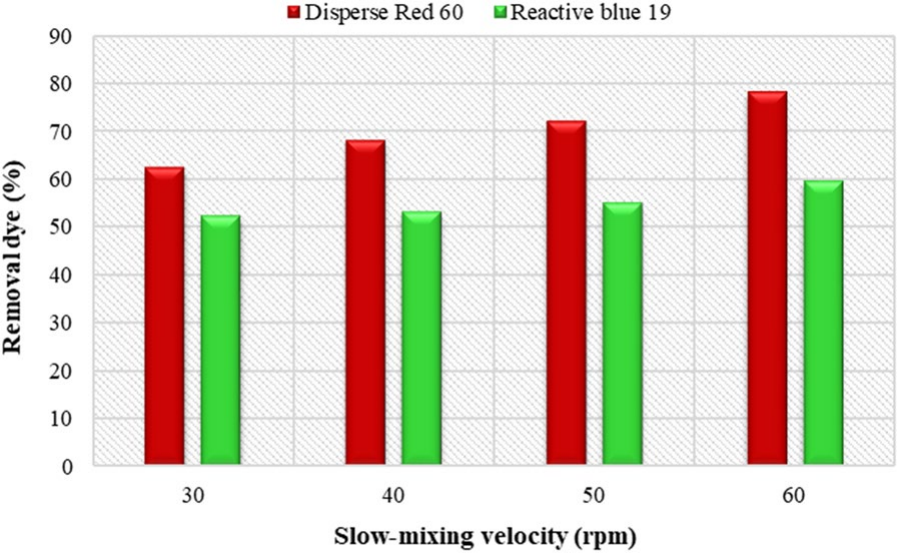
Effect of slow-mixing speed on removal efficiency of Disperse red 60 and Reactive blue 19 dyes. (Initial pH = 11, *Alcea rosea* concentration = 200 mg/L and initial dye concentration = 40 mg/L for Disperse experiments; initial pH = 11, concentration = 250 mg/L and initial dye concentration = 20 mg/L for Reactive experiments)

The results of the effect of slow mixing speed(30 to 60 rpm) on the dye removal efficiency have shown in Fig. 5. The results show that with increasing the slow mixing speed, the dye removal efficiency increases too. So that, the maximum removal efficiency was 78.2% and 59.4% for Dispersed and Reactive dyes at a mixing rate of 60 rpm, respectively. As a result, the mixing speed of 60 rpm was chosen as the optimal mixing speed.

### The effect of slow mixing time

**Fig. 6:**
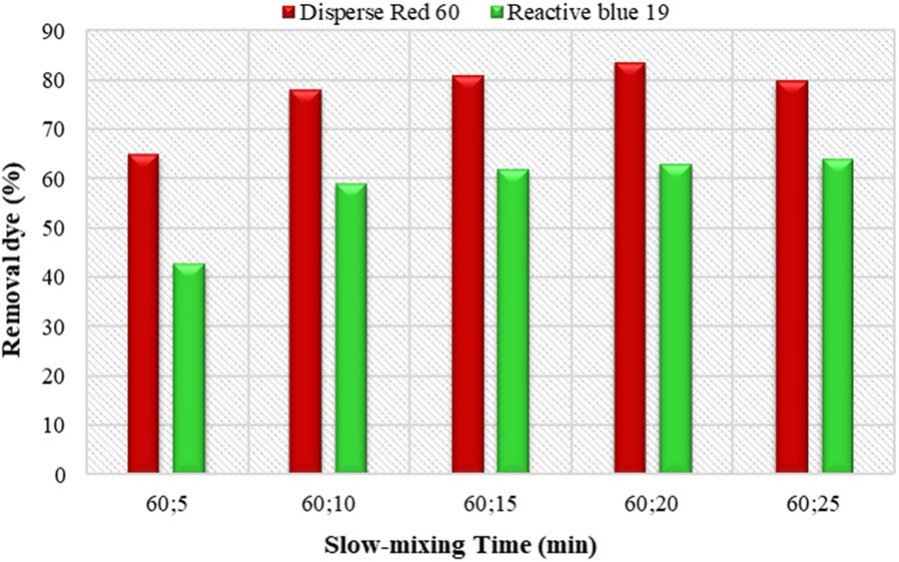
Effect of slow-mixing time on removal efficiency of Disperse red 60 and Reactive blue 19 dyes. (Initial pH = 11, *Alcea rosea* concentration = 200 mg/L and initial dye concentration = 40 mg/L for Disperse experiments; initial pH = 11, concentration = 250 mg/L and initial dye concentration = 20 mg/L for Reactive experiments)

Figure 6 shows the effect of slow mixing time (10–25 min) on the amount of dye removal. As can be seen, the highest removal efficiency of Disperse and Reactive dyes was obtained in a 15 min (81% and 62%, respectively). As the mixing time increases, no significant changes in the dye removal efficiency make. So, 15 min’ time was chosen as the optimal time for slow mixing.

### Effect of settling time

**Fig. 7:**
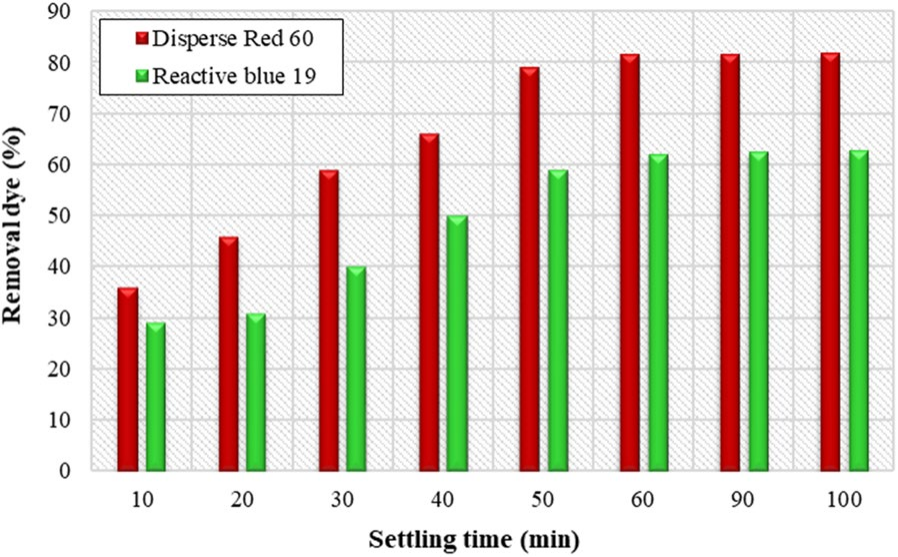
Effect of settling time on removal efficiency of Disperse red 60 and Reactive blue 19 dyes. (Initial pH = 11, *Alcea rosea* concentration = 200 mg/L and initial dye concentration = 40 mg/L for Disperse experiments; initial pH = 11, concentration = 250 mg/L and initial dye concentration = 20 mg/L for Reactive experiments; rapid-mixing speed = 180 rpm; rapid-mixing time = 3 min; slow-mixing speed = 10 rpm; slow-mixing time = 15 min)

During the experiments, the settling time was 60 min, Sampling was performed by examining the effect of different settling times on removal efficiency of the dye (Fig. 7). The results showed that obtained removal efficiency for Disperse and Reactive dyes was 60 min and, at a higher time; effective removal efficiency was not impressive. The results showed that removal efficiency obtained for dispersive and reactive dyes is increasing for 60 min, and at higher rates, removal efficiency is not significant (Fig. 7). As a result, 60 min’ time was chosen as optimal settling time.

### The effect of temperature

**Fig. 8:**
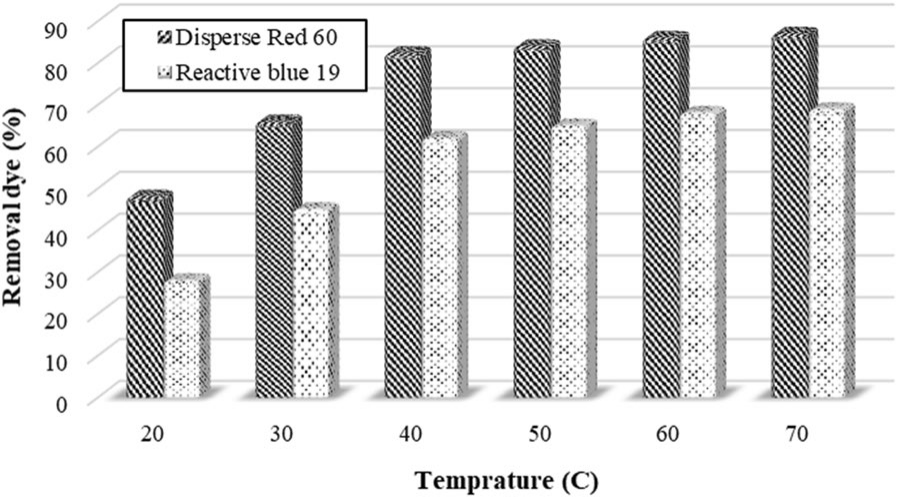
The effect of Temperature on the efficiency of removal of Disperse red 60 and Reactive blue 19 dyes. (Initial pH = 11, *Alcea rosea* concentration = 200 mg/L and initial dye concentration = 40 mg/L for Disperse experiments; initial pH = 11, concentration = 250 mg/L and initial dye concentration = 20 mg/L for Reactive experiments; rapid-mixing speed = 180 rpm; rapid-mixing time = 3 min; slow-mixing speed = 10 rpm; slow-mixing time = 15 min; Sedimentation time = 60 min)

The effect of temperature on the removal efficiency of Disperse red 60 and Reactive blue 19 dyes is shown in Fig. 8. The nature of the curve shows that the removal efficiency of the dye has constantly increased with temperature increasing. The removal efficiency of the dye at temperatures above 60 °C did not significantly increase. So the temperature of 60 °C was chosen as the optimal temperature. The removal efficiency of Disperse red 60 and Reactive blue 19 dyes were 86% and 68%, respectively.

### FT-IR analysis

**Fig. 9:**
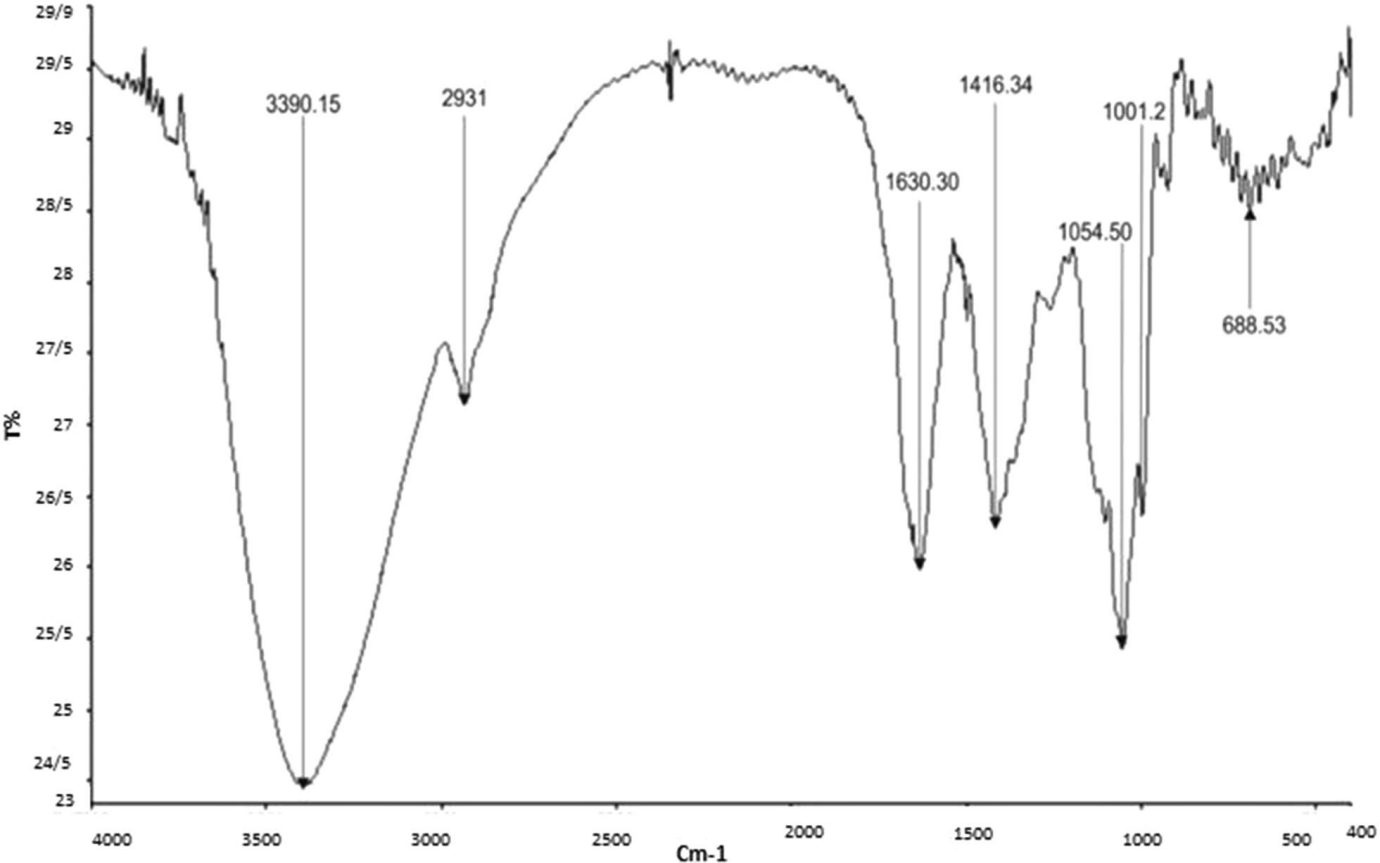
FT-IR Spectrum of *Alce arosea*

The FT-IR spectrum of *Alcea rosea* is shown in Fig. 9. *Alcea rosea* has functional groups indifferent couriers Fig. 9. Peak = 3390 cm^−1^ shows the tensile vibrations of the hydroxyl functional group (O–H), while the peak = 2931 cm^−1^ indicates tensile vibrations of the alkaline group (C–H). Peak = 1630 cm^−1^ shows the tensile vibrations of the carbonyl and peak = (C=O) 1416 cm^−1^, shows the flexural vibrations of the hydroxyl functional group (O–H). Peak = 1054 cm^−1^ and 1001 cm^−1^ show the tensile vibrations of the functional group of alcohols (C–O) and peak = 688 cm^−1^ can be attributed to the flexural vibrations of the C–H group.

## Discussion

pH is a simple parameter but very important that most chemical reactions in the environment are controlled by changing pH value (Sher et al. 2013; Sanghi et al. 2006). The results of the experiments show that *Alcea rosea* root mucilage that is used as a natural coagulant in this study has no significant effect on the removal of Disperse and Reactive dyes in acidic and neutral pH. But in alkaline conditions, the removal efficiency of dye increases. According to Alizadeh et al. the cause of the increased removal efficiency of a Reactive dye under alkaline conditions is probably due to the reaction with hydroxyl ions that forms hydroxyl fluoride (Alizadeh et al. 2014). What was observed during the experiments was the creation of fine and light flocks in acidic pH for both dyes, especially reactive dye, which had a low settling potential. The results of Gohary showed that removal efficiency of Disperse was higher in alkaline conditions than acidic conditions (El-Gohary and Tawfik 2009). Therefore, it is consistent with the results of this study.

The accumulation of particles in the solution can occur through four coagulation mechanisms: (1) double layer compression; (2) sweep flocculation; (3) adsorption and charge neutralization (4) adsorption and interparticle bridging (Shamsnejati et al. 2015). Natural polymer coagulants are generally related to mechanisms (3) and (4) because their long-chain structure can significantly increase the number of un-polarized sites. The presence of a large number of functional groups provides large absorption sites which leading to bridging between particles (Shamsnejati et al. 2015; Freitas et al. 2015). The mechanism of adsorption and charge neutralization refers to the absorption of two particles with opposite charges, while adsorption and interparticle bridging occur when a polymeric coagulant provides a polymer chain that absorbs the particles (Freitas et al. 2015). Alcea mucilage extract as an anionic polysaccharide has a similar mixture of Okra mucilage. It has a galacturonic acid. It is very important that galacturonic acid be predominantly in the polymeric form to provide a chain to absorb particles (Freitas et al. 2015). As a result, the coagulation mechanism of *Alcea rosea* root mucilage is a kind of adsorption and interparticle bridging.

Coagulant concentration is one of the effective factors in the variety of hydrolysis products and coagulation mechanism. Figure 4 shows that removal efficiency of the dye increases with the increasing of coagulant concentration.

However, in concentrations more than optimal value, removal efficiency reduced. The reduction in efficiency can be due to the fact that by adding a coagulant, zeta potential is formed (the potential increases with increasing coagulant concentration and as the potential become closer to zero, removal efficiency will be greater). By adding an optimal amount of coagulant, the Zeta potential becomes zero, but by adding the amount of coagulant, the Zeta potential increases and as a result, it will reduce the removal efficiency (Kim et al. 2004). The results of the study by Subramanian et al. showed that by increasing the concentration of natural coagulant, the removal efficiency of pollutants increased and it continued to a concentration of 1 mg/L and decreased in concentrations above the optimal concentration of removal efficiency (Subramonian et al. 2014) which confirms the result of the present study.

The results of Fig. 4 show that with increasing the initial dye concentration, the removal efficiency of dye increases. But after optimal dye concentration, removal efficiency for Dispersed and Reactive dyes reduces, which can be due to the reduction in the coagulant concentration in the dye solution, because there is no coagulant with increasing dye concentration that can absorb the dye particles. The study by Merzouk et al. showed that by increasing the initial concentration of Disperse red dye(more than 100 mg/L) the removal efficiency reduced (Merzouk et al. 2012). Hence, it is consistent with the results of this study.

Slow mixing step in the coagulation process is essential for the growth and enhancement of clot (Subramonian et al. 2014). In comparison with the coagulant concentration and pH which are independent parameters, the speed and time of mixing depend on parameters such as angular speed (ω = 2π/T) which follows the function of time. “Change of factor at a time” is not used to optimize concentration and pH, but it optimizes the speed and time of mixing. Therefore, the interaction between speed and time should be logical and carried out simultaneously (Ghafari et al. 2010). Zhang et al. investigate the effect of slow mixing on the coagulation process by using poly-aluminum chloride and report that slow mixing time is necessary to achieve optimal coagulation performance (Zhang et al. 2013). The results of the change in speed and time of mixing show that with increasing speed and time of mixing, the efficiency of dye removal increase, in this study, the settling time was measured as a parameter which affect the overall costs and coagulation process efficiency in the purification process (Subramonian et al. 2014). Although, with time increasing, the removal efficiency of Disperse and Reactive dye was increased, but the maximum settling time for both dyes was during the first 60 min. Nevertheless, further increasing of in settling time didn’t have an effect on removal efficiency, so that at 60 min, the removal efficiency was 81.5 and 62% for Disperse and Reactive dyes, respectively. In general, the results of this study showed that Disperse dye removal efficiency was higher than Reactive dye removal that according to Kim and Park, dispersed dye materials due to their low solubility and less ratio of solved chemical oxygen demand (SCOD) to total chemical oxygen demand (TCOD) are easily removed by chemical coagulation compared to reactive dyes (Joo et al. 2007).

The results of similar studies show that coagulation efficiency is directly related to the temperature. The study of Xiao et al. showed that the efficiency of the coagulation process is reduced at low temperatures (Xiao et al. 2009).

## Data Availability

The data of this research are inserted in the present article; other data is available if needed.
